# A low lasing threshold and widely tunable spaser based on two dark surface plasmons

**DOI:** 10.1038/s41598-017-12463-8

**Published:** 2017-10-19

**Authors:** Yanyan Huo, Tianqing Jia, Tingyin Ning, Chaohua Tan, Shouzhen Jiang, Cheng Yang, Yang Jiao, Baoyuan Man

**Affiliations:** 1 Shandong Provincial Key Laboratory of Optics and Photonic Devices, School of Physics and Electronics, Shandong Normal University, Jinan, 250014 China; 2 State Key Laboratory of Precision Spectroscopy, Department of Physics, East China Normal University, Shanghai, 200062 P.R. China

## Abstract

We theoretically demonstrate a low threshold and widely tunable spaser based on a plasmonic nanostructure consisting of two sets of disk-rings (TSDR). The TSDR nanostructure supports two dark surface plasmons (SPs), which are excited simultaneously by two bright SPs at Fano dips. The two dark SPs support lower effective mode volume, higher quality factor and higher Purcell factors. When the dark SPs serve as the pumping and lasing mode of a spaser, the spaser has a lower lasing threshold, a higher pump absorption efficiency and a lower threshold absorbed pump power than the spaser based on a bright SP. In addition, the lasing and pumping wavelengths of the spaser proposed in this article can each be tuned over a very wide wavelength range. Our results should be significant for the development of spasers.

## Introduction

A spaser (surface plasmon amplification by stimulated emission of radiation) supports an ultrasmall and ultrafast coherent optical field^1^, making it a key device for the development of nanoscience and nanotechnology. Early work on spasers primarily focused on designing new nanostructures or materials to suppress losses^2–7^, which is a problem that is faced by all spasers. To our knowledge, most spasers that have been realized are based on bright surface plasmon (SP) modes, which generally have higher lasing threshold. However, amplifying a dark SP with less intrinsic loss is a good approach to solve this problem. The dark SP is the mode that cannot be excited by the incident light directly^8^. As a result, a few studies have considered a spaser based on dark SPs supported by a metamaterial^9,10^. However, a dark SP can be excited effectively by a bright SP at the Fano dip. Dark SPs have been observed in numerous plasmonic systems, such as heterogeneous dimer structures, ring-disk cavities, and dolmen nanostructures^8,11–14^. The dark SPs can be amplified to realize a spaser with a smaller mode volume, a lower threshold and a higher Purcell factor^15,16^.

To date, most nanostructures of spasers only resonate at the lasing mode, as a result, the energy of the incident light cannot be effectively absorbed. This problem can be solved by using a structure resonant at both the lasing mode and pumping mode simultaneously^17^. Because a metallic disk-ring (DR) can support two Fano resonances, two dark SPs, which can serve as the lasing mode and pumping mode of a spaser, can be excited simultaneously^18,19^. Moreover, the DR nanostructure has very good tuning characteristics^20^. The dark SPs supported by DR nanostructures can be manipulated over a broad spectral range. This characteristic makes the DR nanostructure easier to match the absorption peak and emission peak of a gain medium. Recently, enabling the tunability of a spaser has become an interesting research focus; several approaches have been proposed, such as changing the composition and concentration of the gain materials, the width of the nanocavity, the electron-hole pair concentration of the gain materials, and the surrounding environment of metal nanoparticles^6,21–23^. However, only the lasing mode was tuned, and the tuning range was very narrow.

In this Letter, we use a DR composite nanostructure to demonstrate a low threshold and widely tunable spaser based on two dark SPs. The DR composite nanostructure consists of two sets of disk-ring structures (TSDR). Each set can support a dark SP, which can serve as the lasing mode and the pumping mode of a spaser. Dark SPs not only have higher quality factors, lower effective mode volumes and higher Purcell factors but can also be tuned over a very wide wavelength range. Combined with the gain material, the TSDR nanostructure can realize a low threshold and widely tunable spaser based on two dark SPs.

### Numerical model

**Figure 1:**
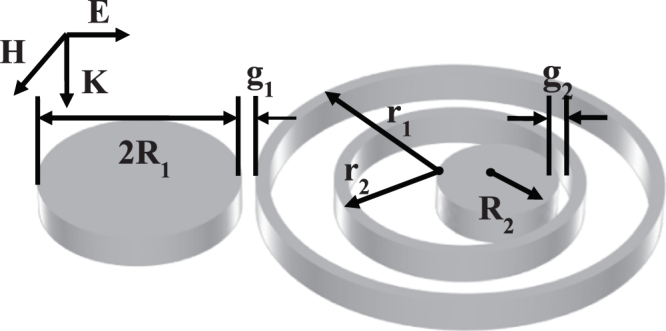
Sketch of the TSDR nanostructure and the incident light.

Figure 1 presents the TSDR nanostructure of the spaser proposed in this article, which consists of two sets of disk-ring structures. One set consists of the outer nanodisk with radius of *R*_1_ and the outer nanoring with radius of *r*_1_, which is called the outer DR nanostructure. Another set consists of the inner nanodisk with radius of *R*_2_ and the inner nanoring with radius of *r*_2_, which is called the inner DR nanostructure. The two nanorings are concentric. The gap between the outer nanodisk and the outer nanoring is *g*_1_ = 20 nm. The centers of the inner nanodisk and inner nanoring are offset by a gap *g*_2_ = 20 nm. The symmetry breaking causes a Fano resonance to appear in the inner DR nanostructure^14^. In this Letter, the wall widths of the two nanorings are all set as 20 nm, and the height of all nanostructures are set at 60 nm to allow for ease of fabrication in an experiment. The TSDR nanostructure is filled with fused silica, with the refractive index *n*_0_ = 1.46. The material for the metallic nanostructure is Ag, with the permeability $\mu =1$ and the complex permittivity sourced from refs^24^. We use the finite element method (Comsol) to investigate the SP characteristics of the TSDR nanostructure. A plane wave irradiates the TSDR nanostructure. When the electric field is parallel to the linked line of the centers of the disk and ring, as shown in Fig. 1, the TSDR structure can support two strong Fano resonances simultaneously. In this case, two dark SPs appear, with each manipulated by a set of the DR nanostructures. In addition, for the spaser based on two SPs, the electric field of the two modes must be spatially overlapped^17,25^. Thus, one nanoring must be set inside another one.

## Results and Discussion

**Figure 2:**
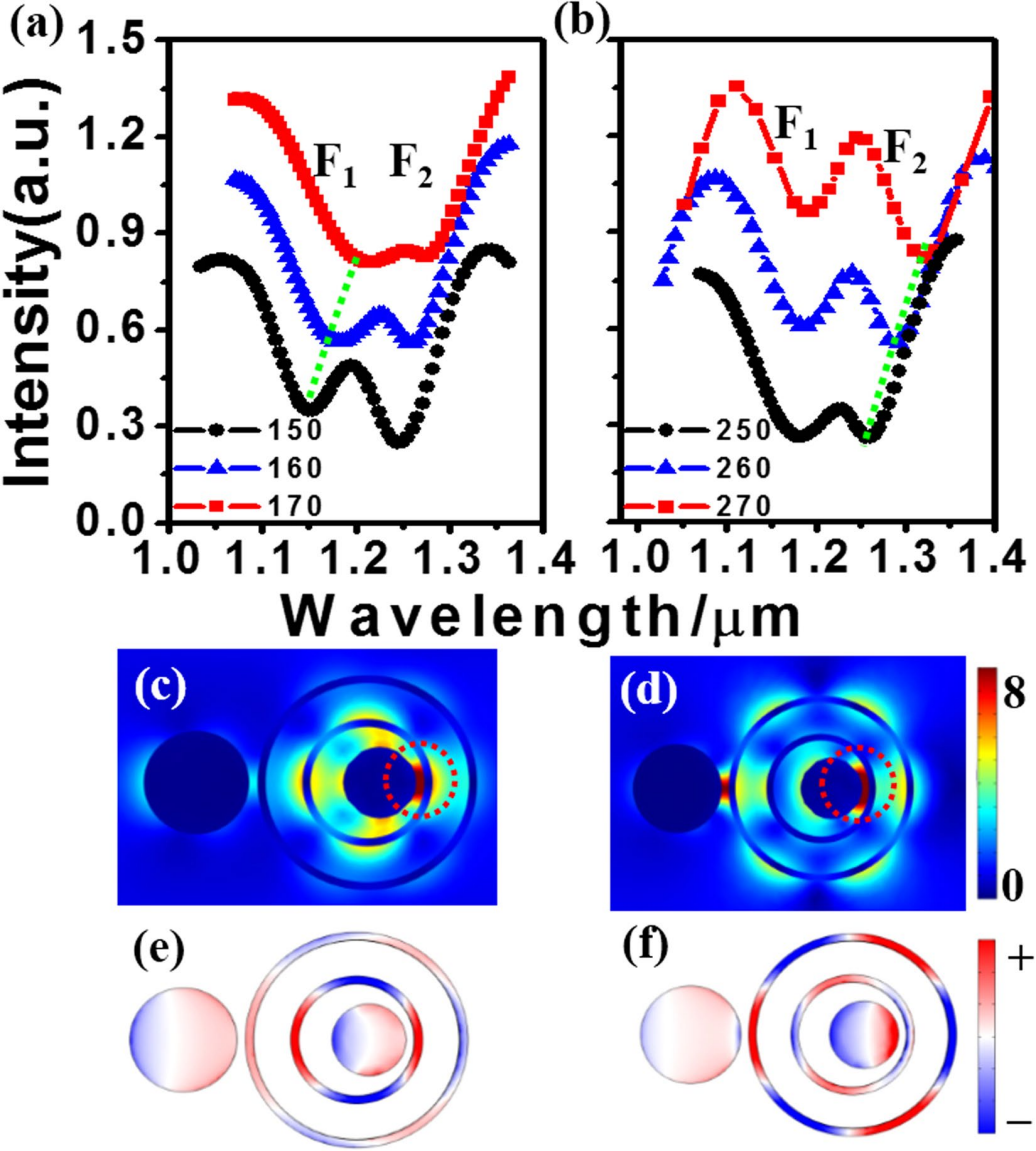
(**a,b**) Scattering spectra of TSDR nanostructures with different radius *r*_2_ of the inner nanoring (**a**) and with different radius *r*_1_ (**b**). The other structure parameters are *R*_1_ = 130 nm, *R*_2_ = 90 nm. (**c–f**) Electric field amplitude in the middle section (**c,d**) and induced surface charges on the top surfaces (**e,f**) at Fano dips F1 (**c,e**) and F2 (**d,f**) of the blue triangle dot line in (**a**).

We first investigate the scattering spectra of the TSDR nanostructure with no gain material. Two evident Fano dips, which are labeled F_1_ and F_2_, appear in every scattering spectrum, as shown in Fig. 2(a) and (b). Figure 2(c–f) show the electric field amplitude and the induced surface charges of the TSDR structure at Fano dips F_1_ and F_2_. It can be seen that the quadrupole resonance of inner nanoring and the octupole resonance of outer nanoring are excited. However, for a single ring, the multipolar resonances cannot be excited directly by the plane wave shown in Fig. 1 ^20^. We call the multipolar resonances of the nanoring the dark SPs. These dark multipolar resonances can be excited by bright mode at the Fano dips. The quadrupole resonance of the inner ring is excited by the dipolar resonance of the inner disk at Fano dip F_1_. The octupolar resonance of the outer nanoring is excited by the dipolar resonance of the outer disk at Fano dip F_2_^19^. The resonant wavelength of each dark resonance can be manipulated by changing the sizes of the set of two nanorings. This feature allows the TSDR nanostructure to have a very good tuning characteristic.

Figure 2(a) and (b) present the good tunability of the TSDR nanostructure. In Fig. 2(a), we keep the radius of outer nanoring *r*_1_ = 250 nm unchanged and adjust the radius *r*_2_ of the inner nanoring from 150 to 170 nm, Fano dip F_1_ is tuned from 1149 to 1217 nm, as shown by the green dotted line in Fig. 2(a). Fano dip F_2_ redshifts slightly because of the stronger interaction of inner and outer ring^19^. If we keep *r*_2_ = 160 nm unchanged and adjust the radius *r*_1_ of outer nanoring from 250 to 270 nm, Fano dip F_2_ can be tuned from 1181 to 1321 nm, as shown by the green dotted line in Fig. 2(b). However, Fano dip F_1_ remains essentially unchanged because of the weaker interaction of the inner and outer nanoring as *r*_1_ increases. From the above discussion, we can see that the two Fano dips can be tuned arbitrarily, i.e., the relative and absolute wavelength of the two dark SPs can be tuned arbitrarily. This tuning makes it easier to match the two dark SPs the lasing and pumping mode of a spaser. In addition, the electric fields of the two dark SPs not only can be enhanced by approximately 8 times but also have spatial overlap. It is especially important that they have a same spatial region with the strongest electric field, as shown by the red dotted circle in Fig. 2(c) and (d). This region will be the lasing region because of the stronger electric field. As a result, this TSDR nanostructure is appropriate for realizing a widely tunable spaser based on two dark SPs.

**Figure 3:**
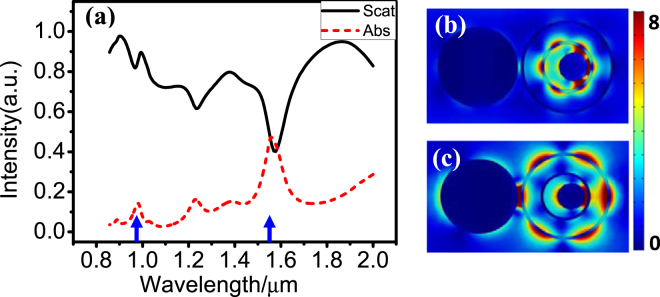
(**a**) The scattering spectra and absorption spectra of the TSDR nanostructure with 980 and 1550 nm dark SPs. (**b,c**) The electric field amplitude in the middle section at the Fano dips of 980 nm (**b**) and 1550 nm (**c**).

The two dark SPs can be tuned to easily match the absorption peak and emission peak of a gain medium. If we select the semiconductor (e.g., InGaAsP) layer or quantum well structure (e.g., InGaAsN/InP or InGaAsP/InP) to act as the gain medium layer, the absorption peak is at 980 nm, and the emission peak is at 1550 nm. We can adjust the radii of every nanodisk and nanoring to make the two Fano dips, i.e., two dark SPs, close to 980 nm and 1550 nm. Figure 3 shows the scattering and absorption spectra of the TSDR nanostructure after careful adjustment. When *R*_1_ = 280 nm, *R*_2_ = 100 nm, *r*_1_ = 325 nm, and *r*_2_ = 181 nm, two Fano dips appear in the scattering spectra that are very close to 980 and 1550 nm, as labeled by the blue arrows. Figure 3(b) and (c) show the electric field distribution of the two dark SPs at 980 and 1550 nm. The dark SP at 1550 nm is still the octupole resonance of the outer nanoring, and the dark SP at 980 nm is the octupole resonance of inner nanoring instead of the original quadrupolar resonance, as shown in Fig. 2(c), because we must reduce the radius of the inner nanoring to make the dark SP blueshift but cannot decrease it without limit. Another Fano dip appears at 1232 nm, which originates from the outer nanoring’s hexadecapole resonance.

The spontaneous or stimulated emission rate of an atom or ion can be enhanced by a nanocavity, with the degree of enhancement given by the Purcell factor ${F}_{p}=3Q{\lambda }^{3}/(4{\pi }^{2}{V}_{eff}{n}_{eff}^{3})$, where *λ* is the wavelength of dark SP excited at the Fano dip, and *n*_*eff*_ = 1.46 is the effective refractive index of the gain material layer^26^. The quality factor is estimated by *Q* = *λ/∆λ*, where *∆λ* is the spectral line width of the Fano dip. The effective mode volume *V*_*eff*_ is calculated using the formula: ${V}_{eff}={\int }_{V}W(r){d}^{3}r/\max \,[W(r)]$, where $W(r)$ is the energy density calculated by $W(r)=\frac{1}{2}\{\mathrm{Re}[d\varepsilon (r)\omega /d\omega ]{|E(r)|}^{2}+{\mu }_{0}{|H(r)|}^{2}\}$, $\varepsilon (r)$ is the dielectric constant at position *r*, ${\mu }_{0}$ is the vacuum magnetic permeability, ${|E(r)|}^{2}$ and ${|H(r)|}^{2}$ are the electric and magnetic field distributions, respectively, and *V* is a quantization volume that includes the resonator and the radiation zone of the resonance mode of the TSDR nanostructure and the radiation zone of the resonance mode of the TSDR nanostructure. The quality factors, effective mode volumes, and Purcell factors of the dark SP at 980 nm (1550 nm) are calculated as 40 (28), $1.2\times {10}^{-2}{(\lambda /{n}_{eff})}^{3}$ ($1.77\times {10}^{-3}{(\lambda /{n}_{eff})}^{3}$), and 2.53 × 10^2^ (1.2 × 10^3^), respectively. These characteristics are expected to enable the spaser to have a low lasing threshold and a low threshold absorbed pump power^17^.

**Figure 4:**
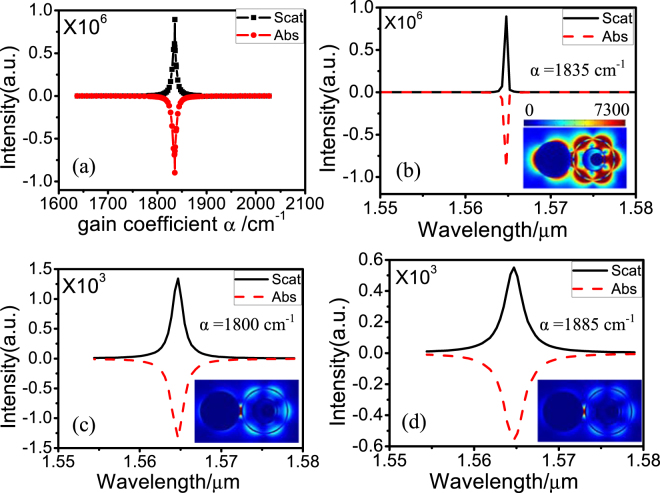
(**a**) Scattering and absorption spectra calculated at 1550 nm for the TSDR nanostructure supported dark SP mode with optical gains. The structural parameters are same as those used in Fig. 3(b–d). The scattering and absorption spectra calculated at the threshold of α = 1835 cm^−1^ (**b**), α = 1800 cm^−1^ (**c**) and α = 1835 cm^−1^ (**d**). Insets: the electric field amplitude in the middle section of the dark SP at 1550 nm at each gain coefficient in (**b–d**).

In the following, we demonstrate that the TSDR nanostructure with resonances at 980 and 1550 nm can act as a low lasing threshold spaser. A complex refractive index *n*_*g*_ = 1.46 -i*k* is used to model the dielectric containing active materials. *k* is the gain coefficient, which is related to the gain coefficient $\alpha$ by $\alpha =2\pi |k|/\lambda$ ^27^. Figure 4(a) shows the calculated scattering and absorption spectra of the dark SP at 1550 nm as a function of the optical gain. With increase of the optical gain from α = 0 to α = 1820 cm^−1^, the intensity of scattering gradually increases [black dotted line in Fig. 4(a)], while the absorption gradually decreases and reaches the value of zero at α = 50 cm^−1^, and then the absorption becomes negative [red dotted line in Fig. 4(a)], indicating that the metallic Ohmic loss begins to be compensated by the gain media. When the gain is α = 1835 cm^−1^, the intensity of scattering reaches the maximum values, and the absorption reaches the minimum value, with the extinction close to zero. It has been shown previously that a zero-level net optical gain can give rise to the maximum spasing efficiency^28^. Thus, the optimal optical gain of α = 1835 cm^−1^ is viewed as a lasing threshold. When the gain coefficient reaches the threshold, the energy provided by the gain and the loss of the system are under dynamic balance^15^. The gain coefficient of the InGaAsN/InP and InGaAsP/InP quantum wells can exceed 2000 cm^−1^ at room temperature^29,30^. The quantum wells can provide the gain energy for the dark SP at 1550 nm at room temperature.

The calculated scattering and absorption spectra as a function of the incident light wavelength are shown in Fig. 4(b–d). When the optical gain α = 1835 cm^−1^, i.e., at the lasing threshold, the scattering and absorption of the dark SP resonance of 1550 nm can reach high magnitudes of 9.0 × 10^5^, the linewidths can be significantly narrowed to 0.4 nm, and the electric field amplitude in the middle section is greatly enhanced, which can be enhanced by 7.3 × 10^3^, as shown in the inset of Fig. 4(b). When the gain α > 1835 cm^−1^ or α < 1835 cm^−1^, the scattering intensity and the electric field amplitude decreases greatly, whereas the linewidth increases greatly, as shown in Fig. 4(c) and (d). When α < 1835 cm^−1^, the energy provided by the gain media cannot compensate the loss of the TSDR nanosystem; however, when α > 1835 cm^−1^, the dynamic balance of the gain and loss is broken, and the lasing disappears.

**Figure 5:**
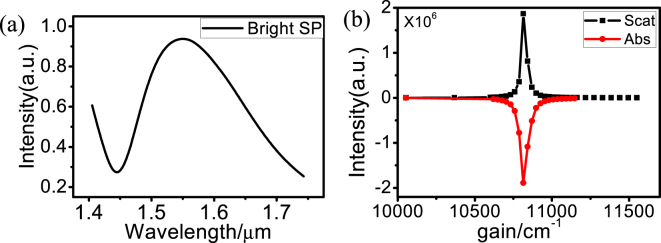
(**a**) Scattering spectrum calculated at 1550 nm for the TSDR nanostructure supported bright SP mode with optical gains. The structural parameters are *R*_1_ = 195 nm, *R*_2_ = 90 nm, *r*_1_ = 350 nm, and *r*_2_ = 185 nm. (**b**) The scattering and absorption spectra calculated at the threshold of α = 10800 cm^−1^.

We change the size of the outer nanodisk and nanoring to shift a bright SP to the wavelength of 1550 nm, as shown in Fig. 5(a). The bright SP mode can also be amplified to realize a spaser, as shown in Fig. 5(b). Its lasing threshold reaches up to 10800 cm^−1^, which is 6 times higher than that of the spaser based on the dark mode, and the gain cannot be provided by the InGaAsN/InP or InGaAsP/InP quantum wells at room temperature. As a result, a spaser with lower lasing threshold can be realized by amplifying a dark SP, which is enabled by the lower intrinsic loss of the dark SP mode.

In order to improve the pump absorption efficiency, a dark SP is used to act as the pumping mode of the spaser. The maximum laser output power can be obtained at the optimized pump absorption efficiency^31^. The absorption efficiency is defined as the power absorption of the nanostructure divided by the incident light output^31^. We compare the pump absorption efficiency when the dark SP or the bright SP serves as the pumping mode. The pump absorption efficiency of the dark SP can be enhanced by 4.2 times compared to that of the bright SP. The bright SP at 980 nm is obtained when the nanodisk is concentrically placed in the inner nanoring. At the Fano dips, the bright mode is excited by two pathways: |L >  → |B > and |L >  → |B >  → |D > → |B > , where |L > , |B > , and |D > are the incident light, bright SP, and dark SP mode, respectively. The destructive interference of the two pathways leads to the appearance of the Fano resonance and the suppression of the bright mode, i.e., only the dark SP mode appears. The energy absorbed by the nanostructure-supported Fano resonance transfers to the dark SP mode. However, for the concentric inner DR nanostructure, the bright SP mode is only excited by one pathway: |L >  → |B > . As a result, the pump absorption efficiency of the dark SP mode is higher than that of the bright SP. It can also be demonstrated by the electric field amplitude^32^. The electric field amplitude of the dark mode can be enhanced by 28.7 times, whereas the bright mode is only enhanced by 12.8 times. The threshold absorbed pump power dependence on the doping density and the nanostructure must be considered. If we assume the doping densities are the same for the nanostructure resonant with the 980-nm dark mode and bright mode, then the threshold absorbed pump power is inversely proportional to the *Q* factor and is proportional to the group velocity^17^. The *Q* factor of the dark and bright SP mode is 40 and 6.4, respectively. In other words, more metallic losses must be compensated by a higher pumping power for a bright SP mode. In addition, the group velocity will slow down at the Fano dip^10^^.^ As a result, the threshold absorbed pump power of the spaser based on the dark SP is lower than that of the spaser based on the bright SP.

**Figure 6:**
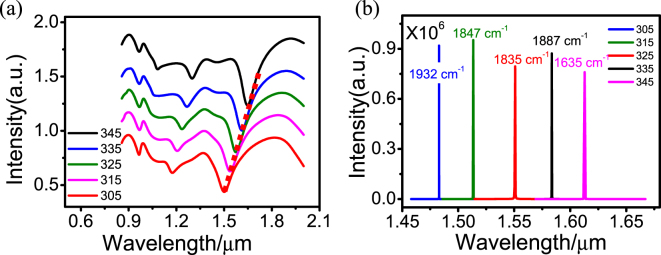
(**a**) Scattering spectra of the TSDR nanostructure with different radius *r*_1_ of the outer nanoring, with the other structural parameters the same as those used in Fig. 3. (**b**) The scattering spectra of the dark SPs of the outer nanoring calculated at their respective lasing threshold. The lasing threshold of each SP is labeled in figure (**b**).

When the lasing and pumping mode are tuned over a wide wavelength range, the lasing can also be realized at room temperature. Figure 6 describes the good tunability of the lasing mode. We keep the 980-nm pumping mode unchanged, i.e., maintain the inner nanoring’s octupole Fano resonance and adjust the radius *r*_1_ of outer nanoring from 305 to 345 nm, the lasing mode based on the outer nanoring’s octupole resonance is from 1.49 to 1.64 µm, as shown in Fig. 6(a) with the red dotted line. If we do not consider the limit of the Ag dielectric constant, we can further increase *r*_1_ such that the lasing wavelength can be tuned to the middle infrared wavelength range, even to the far infrared wavelength range. Figure 6(b) shows the lasing modes amplified by stimulated emission of radiation. We can see every dark SP can be amplified, and all the line widths are 1–2 nm. The threshold of each lasing mode is slightly different, as labeled in Fig. 6(b); they are all lower than that of the spaser based on the bright mode. The InGaAsN/InP or InGaAsP/InP quantum wells can provide the gain energy for these lasing dark modes at room temperature.

**Figure 7:**
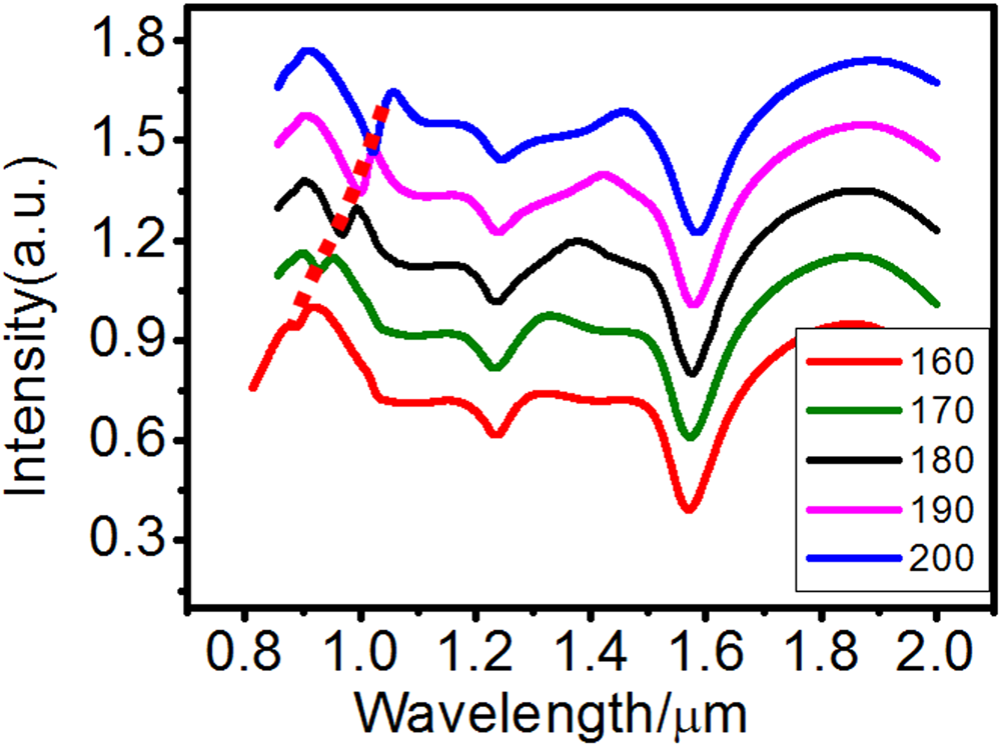
Scattering spectra of the TSDR nanostructure with different radius *r*_2_ of the inner nanoring, with the other structural parameters the same as those used in Fig. 3(b).

Figure 7 shows the good tunability of the pumping mode. We keep the 1550 nm lasing mode unchanged, i.e., maintain the wavelength of outer nanoring’s octupole dark SP and adjust the radius *r*_2_ of the inner nanoring from 160 to 200 nm, the pumping mode based on the inner nanoring octupole resonance is tuned from 0.88 to 1.03 µm, as shown in Fig. 7 with the red dotted line. The scattering of the pumping mode becomes smaller as *r*_2_ decreases, thereby reducing the threshold absorbed pump power; however, the lasing threshold remains unchanged at approximately 1840 cm^−1^. Other Fano dips, which originate from the higher order dark SPs of the outer nanoring and the inner nanoring, appear in Figs 6 and 7. The lasing mode and pumping mode of the TSDR nanostructure have a wide tunable range. If we want to realize a widely tunable spaser in an experiment, we require gain materials with a broad band or we must change the bandgap of the gain materials by some methods, for example, changing the composition, the electron-hole pair concentration and the concentration of the gain materials^6,21,22^.

## Conclusions

In summary, we theoretically realized a low threshold and widely tunable spaser based on two dark SPs of a TSDR nanostructure. The two dark SPs, which are excited by the bright SP mode at two Fano resonances, have higher quality factor, lower effective mode volume and higher Purcell factor. When the dark SPs serve as the lasing mode and pumping mode of a spaser, the spaser has a lower lasing threshold of α = 1835 cm^−1^, which is much lower than that of a spaser based on the bright mode. The lasing threshold is also lower than that of a spaser based localized surface plasmon; for example, a high lasing threshold value α = 7080 cm^−1^ is required for a semishell-capped system^33^. In addition, the higher pump absorption efficiency and lower threshold absorbed pump power of the proposed spaser enhances the utilization of incident light. Moreover, the pumping and lasing wavelengths of the spaser can each be tuned over a broad spectral range because every mode is manipulated by different set of DR structures in the TSDR nanostructure.
